# Machine Learning-Based Random Forest to Predict 3-Year Survival after Endovascular Aneurysm Repair

**DOI:** 10.5761/atcs.oa.25-00036

**Published:** 2025-05-15

**Authors:** Toshiya Nishibe, Tsuyoshi Iwasa, Seiji Matsuda, Masaki Kano, Shinobu Akiyama, Shoji Fukuda, Masayasu Nishibe

**Affiliations:** 1 Department of Medical Management and Informatics, Hokkaido Information University, Ebetsu, Hokkaido, Japan; 2 Department of Cardiovascular Surgery, Tokyo Medical University, Tokyo, Japan; 3 Department of Surgery, Eniwa Midorino Clinic, Eniwa, Hokkaido, Japan

**Keywords:** abdominal aortic aneurysm, endovascular aneurysm repair, machine learning, random forest, feature importance

## Abstract

**Purpose:** Endovascular aneurysm repair (EVAR) is widely used to treat abdominal aortic aneurysms (AAAs), but mid-term survival remains a concern. This study aims to develop a machine learning-based random forest model to predict 3-year survival after EVAR.

**Methods:** A random forest model was trained using data from 176 EVAR patients, of whom 169 patients were retained for analysis, incorporating 23 preoperative and perioperative variables. Model performance was evaluated using 5-fold cross-validation.

**Results:** The model achieved an area under the receiver-operating characteristic curve (AUC) of 0.91, with an accuracy of 81.1%, a sensitivity of 81.6%, a specificity of 80.9%, and an F1 score of 0.66. Feature importance analysis identified poor nutritional status (geriatric nutritional risk index <101.4), compromised immunity (neutrophil-to-lymphocyte ratio >3.19), chronic kidney disease (CKD), octogenarian status, chronic obstructive pulmonary disease (COPD), small aneurysm size, and statin use as the top predictors of 3-year mortality. The average values of the AUC, accuracy, sensitivity, specificity, and F1 score across the 5-folds were 0.76 ± 0.10, 73.9 ± 5.8%, 60.4 ± 1.9%, 77.8 ± 0.7%, and 0.59 ± 0.17, indicating consistent performance across different data subsets.

**Conclusions:** The random forest model effectively predicts 3-year survival after EVAR, indicating key factors such as poor nutritional status, compromised immunity, CKD, octogenarian status, COPD, small aneurysm size, and statin use.

## Introduction

Endovascular aneurysm repair (EVAR) has revolutionized the treatment of abdominal aortic aneurysms (AAAs) by offering a minimally invasive alternative to open surgery. The benefits of EVAR include reduced operative time, lower perioperative morbidity, and shorter hospital stays.^1)^ However, despite these initial benefits, early postoperative mortality remains a concern, particularly within the first 2 to 3 years after the procedure.^2,3)^ EVAR reduces perioperative surgical risks, but patients with significant comorbidities continue to face an elevated risk of short- to mid-term mortality.^2)^ Specific patient characteristics that contribute most to early mortality remain incompletely understood, underscoring the need for improved risk stratification and predictive models.^4,5)^

Traditional statistical models, such as logistic regression, have long been used for risk stratification. However, their predictive power is limited by assumptions of linearity and variable independence, which restrict their ability to capture complex interactions among multiple risk factors.^6)^ In contrast, machine learning techniques, particularly random forests, offer a more sophisticated approach to clinical prediction. By using ensemble learning, random forests can analyze high-dimensional data, uncover complex relationships among variables, and generate more accurate and reliable predictions.^7)^ Additionally, random forests enhance understanding of individual variable contributions by ranking feature importance, providing insights into the impact of each predictor on patient outcomes.^8)^

Recent studies have demonstrated the potential of machine learning models, particularly random forests, in predicting survival after AAA repair.^9,10)^ Thompson et al. reported that a random forest classifier achieved high accuracy in predicting 2-year survival following elective AAA repair, highlighting its value in clinical decision-making.^9)^ Lareyre and Teraa discussed both the strength and limitations of machine learning-based risk stratification in AAA patients, emphasizing the need for external validation.^10)^ Together, these studies can provide a strong foundation for further exploration of machine learning’s role in outcome prediction after EVAR.

The purpose of this study is to evaluate the predictive performance of random forest models in determining 3-year survival after EVAR and to assess feature importance in identifying key predictors of survival. By analyzing a comprehensive set of preoperative and perioperative variables, this study aims to demonstrate the clinical utility of machine learning in optimizing patient management and improving mid-term survival outcomes.

## Materials and Methods

### Patients

This retrospective study analyzed 176 patients who underwent elective EVAR for AAA at Tokyo Medical University Hospital between October 2013 and July 2018. The cohort is identical to the one used in a previous study.^11)^ The indications for EVAR were symptomatic AAA, asymptomatic AAA ≥50 mm in diameter, or rapidly expanding AAA (5 mm in 6 months), based on the Guideline for the Diagnosis and Treatment of Aortic Aneurysm and Aortic Dissection issued by Japanese Circulation Society.^12)^ Small aneurysms (<50 mm) treated at the patient’s request were also included. Patients with ruptured or mycotic AAA, as well as those with isolated common or internal iliac artery aneurysms, were excluded. Patients were selected from a prospectively maintained database and followed for 3 years postoperatively; those lost to follow-up within this period were excluded from the analysis.

At the initial consultation, all patients were asked whether they were willing to provide written informed consent for their clinical data to be used in scientific presentations or publications. The procedures followed were in accordance with the Ethical Guidelines for Medical and Biological Research Involving Human Subjects (enacted on March 23, 2021 by the Japanese Government) and the Declaration of Helsinki of 1975, as revised in 2008. This study was approved by the Clinical Research Committee of Tokyo Medical University, where it was conducted (TS2020-0388, January 15, 2021).

### Dataset

The dataset comprised demographic factors (octogenarians, female sex, and smoking); poor nutritional status, defined as geriatric nutritional risk index [GNRI] <101.4^13,14)^; comorbidities (hypertension, dia-betes mellitus, dyslipidemia, ischemic heart disease, cerebrovascular disease, chronic obstructive pulmonary disease [COPD], and chronic kidney disease [CKD], defined as estimated glomerular filtration rate <60 mL/min/1.73 m^2^); medications (statins, β-blockers, and antiplatelet drugs); aneurysm morphology (small aneurysm size, defined as diameter <50 mm); surgical data (graft type [expanded polytetrafluoroethylene vs. polyester], outside the instruction for use [IFU], operative type II endoleak, internal iliac artery embolization, and reinterventions); inflammation, defined as C-reactive protein [CRP] >0.3 mg/dL; and compromised immunity, defined as neutrophil-to-lymphocyte ratio [NLR] >3.19.^15)^

The GNRI, which combines albumin and body mass index, was proposed by Bouillanne et al. as an objective nutritional risk index.^13)^ The NLR, calculated by dividing the absolute neutrophil count by the absolute lymphocyte count, is a useful marker for assessing immune-related clinical outcomes in patients with various diseases. Our previous reports set the cutoff values for the GNRI and NLR at 101.4 and 3.19, respectively.^14,15)^

### Survival

Patients were followed through the database until death or the end of the follow-up period in November 2021, according to institutional protocol.^14,15)^ Survival status was confirmed through outpatient visits, letters, or phone calls when clinic visits were not possible.

### Statistical analysis

Data are presented as mean ± standard deviation for continuous variables. Prism 9 for Mac OS X (GraphPad Software Inc., La Jolla, CA, USA) and Mac Toukeikaiseki (Esumi, Tokyo, Japan) were used for statistical analysis. Categorical data were analyzed using the Pearson χ^2^ test or Fisher’s exact probability test.

### Random forest models

The random forest classifier was implemented using Python 3.7 and the scikit-learn library. The dataset was preprocessed by normalizing continuous variables and encoding categorical variables as needed. A total of 23 variables were included in the model, selected based on clinical relevance and prior research findings. Feature importance was assessed using the Gini impurity criterion, which quantifies each variable’s contribution to reducing classification uncertainty. This metric helps identify the input variables that most strongly influence the model’s predictions, thereby enhancing interpretability and informing feature selection. To balance model performance and complexity, features accounting for up to 70% of the cumulative importance were retained. This approach helps preserve the most predictive variables while minimizing the risk of overfitting.

Model training and evaluation were conducted using 5-fold cross-validation, where the dataset was randomly divided into 5 subsets. Four subsets were used for training, while the remaining subset served as the validation set. This process was repeated 5 times to enhance generalizability. To optimize model performance and enhance its discriminative ability, hyperparameter tuning was performed using randomized search optimization with the area under the receiver-operating characteristic curve (AUC) as the evaluation metric. Key parameters such as the number of trees, maximum depth, and minimum samples per split were adjusted. Performance metrics included AUC, accuracy, sensitivity, specificity, and F1 score. These metrics were computed for each fold and averaged to derive the final model performance estimates.

## Results

### Patients, events, and follow-up

Of the 176 patients initially included, 169 were retained in the study, and 7 were excluded based on the exclusion criteria because they were lost to follow-up within 3 years. The cohort consisted of 143 males (84.6%) and 26 females (15.4%) with a mean age of 77.2 ± 7.8 years (range: 51–89 years). Of these, 131 (77.5%) survived more than 3 years, and 38 (22.5%) died within 3 years. None of the deaths were related to aneurysms; 16 were due to cancer, 8 to pneumonia, 4 to cerebrovascular disease, 2 to kidney disease, and 3 to heart disease. Five cases were unexplained but unrelated to the aneurysm.

### Univariable analysis

**Table 1** shows comparisons of adverse or favorable predictors of early mortality between patients who survived for more than 3 years and those who died within 3 years. Patients who died within 3 years had significantly poorer nutritional status (77.5% vs. 42.0%, p <0.001) and compromised immunity (63.5% vs. 26.7%, p <0.001). They also had significantly higher rates of CKD (65.8% vs. 41.2%, p = 0.008), octogenarian status (63.2% vs. 38.9%, p = 0.008), COPD (44.7% vs. 22.1%, p = 0.006), and active cancer (42.1% vs. 22.9%, p = 0.018), along with a significantly lower rate of small aneurysm size (26.3% vs. 47.3%, p = 0.021).

**Table 1:** Predictors of early mortality between patients who survived more than 3 years and those who died within 3 years

Variables	Died (n = 38)	Survived (n = 131)	p value
Age (years)	82.0 ± 5.0	75.8 ± 7.7	<0.001*
Octogenarian status (≥80 years)	24 (63.2)	51 (38.9)	0.008*
Female	2 (5.3)	24 (18.3)	0.071
GNRI	95.8 ± 8.7	102.0 ± 9.4	<0.001*
Poor nutritional status (GNRI <101.4)	31 (81.6)	55 (42.0)	<0.001*
Smoking	24 (63.2)	85 (64.9)	0.845
Concomitant disease			
Hypertension	31 (81.6)	101 (77.1)	0.557
Dyslipidemia	16 (42.1)	62 (47.3)	0.570
Diabetes mellitus	6 (15.8)	30 (22.9)	0.346
CKD	25 (65.8)	54 (41.2)	0.008*
COPD	17 (44.7)	29 (22.1)	0.006*
Cerebrovascular disease	17 (44.7)	41 (31.3)	0.125
Ischemic heart disease	21 (55.3)	86 (65.6)	0.596
Active cancer	16 (42.1)	30 (22.9)	0.018*
Medication			
Antiplatelet drugs	11 (28.9)	57 (43.5)	0.107
Statins	14 (36.8)	59 (45.0)	0.898
β-blockers	10 (26.3)	51 (38.9)	0.154
Aneurysm sac diameter (mm)	54.3 ± 8.1	51.9 ± 7.8	0.058
Small aneurysm size (diameter <50 mm)	10 (26.3)	62 (47.3)	0.021*
Operative data			
Graft type			
Polyester	15 (39.5)	36 (27.5)	0.156
Expanded polytetrafluoroethylene	23 (60.5)	95 (72.5)	Reference
Outside IFU	6 (15.8)	20 (15.3)	0.937
Operative type II endoleak	6 (15.8)	31 (23.7)	0.301
Internal iliac artery embolization	6 (15.8)	13 (10.0)	0.314
Reintervention	4 (10.5)	14 (10.7)	>0.999
CRP			
Inflammation (CRP >0.3 mg/dL)	9 (23.7)	31 (23.7)	0.998
NLR	3.59 ± 1.64	2.85 ± 1.34	0.007*
Compromised immunity (NLR >3.19)	23 (60.5)	35 (26.7)	<0.001*

*Significant.

Unless indicated otherwise, data are presented as mean ± SD or n (%).

GNRI: geriatric nutritional risk index; CKD: chronic kidney disease; COPD: chronic obstructive pulmonary disease; IFU: instruction for use; CRP: C-reactive protein; NLR: neutrophil-to-lymphocyte ratio

### Feature importance

**Fig. 1:**
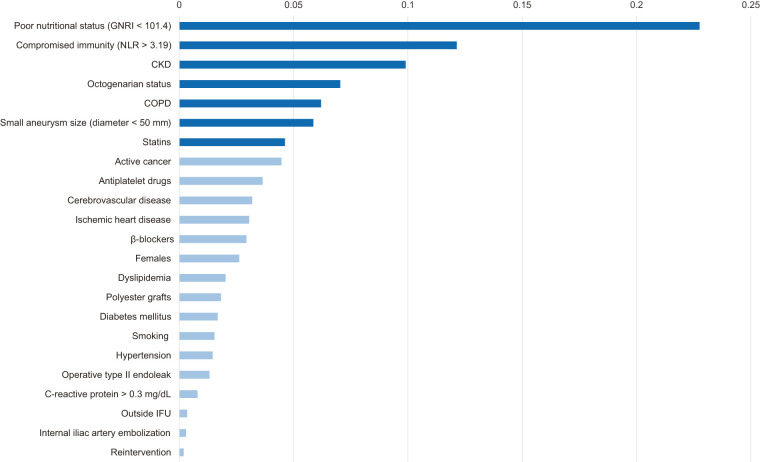
Key predictors of 3-year survival identified by the random forest model. The key predictors are represented by the 7 dark blue bars. GNRI: geriatric nutritional risk index; CKD: chronic kidney disease; COPD: chronic obstructive pulmonary disease; IFU: instruction for use; NLR: neutrophil-to-lymphocyte ratio

The random forest model identified several key predictors, both adverse and favorable, of 3-year survival (**Fig. 1**). The most important predictor was poor nutritional status, defined by GNRI <101.4, with an importance score of 0.2276. Other notable predictors included compromised immunity (NLR >3.19, importance score 0.1214), CKD (0.0990), octogenarian status (0.0705), COPD (0.0620), small aneurysm size (0.0587), and statin use (0.0462).

### Model performance

**Fig. 2:**
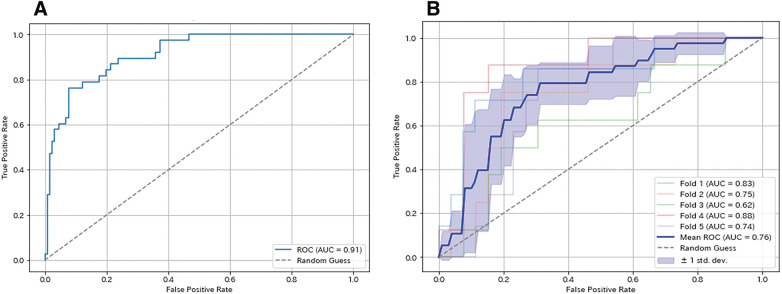
Receiver-operating characteristic curve (ROC) for predicting 3-year mortality following endovascular abdominal aortic aneurysm repair using random forest analysis. AUC, area under the ROC. (**A**) Entire dataset and (**B**) 5-fold cross-validation.

The random forest model demonstrated high predictive performance in assessing 3-year survival outcomes after EVAR. The parameters that resulted in the highest AUC value after performing a random search with 100 trials were as follows: maximum depth of the trees, 13; number of trees, 480; minimum number of samples required to split an internal node, 12; minimum number of samples required to be at a leaf node, 9; and class weight adjustment for balancing the class distribution. Consequently, with an AUC of 0.91, the model showed high discriminative ability (**Fig. 2A**). It also achieved an accuracy of 81.1%, a sensitivity of 81.6%, a specificity of 80.9%, and an F1 score of 0.66, underscoring its ability to accurately identify both survival and non-survival cases.

To further validate the model’s stability and generalizability, 5-fold cross-validation was conducted. The dataset was divided into 5 subsets, each serving as both the training and test set. The average values of AUC, accuracy, sensitivity, specificity, and F1 score across the 5-folds were 0.76 ± 0.10, 73.9 ± 5.8%, 60.4 ± 1.9%, 77.8 ± 0.7%, and 0.59 ± 0.17, respectively, indicating consistent performance across different data subsets. The variability in performance across folds is illustrated by the shaded region of the receiver-operating characteristic curve (**Fig. 2B**), which highlights the model’s robustness despite data variability.

## Discussion

This study demonstrates that the random forest model is a reliable and effective tool for predicting 3-year survival outcomes following EVAR. The model achieved a high AUC of 0.91, an accuracy of 81.1%, a sensitivity of 81.6%, a specificity of 80.9%, and an F1-score of 0.66, reflecting its strong predictive performance. These results underscore the model’s ability to accurately differentiate between patients who will survive for 3 years and those who will not, providing valuable insights into long-term survival after EVAR.

Random forest is an ensemble learning method that combines multiple decision trees to make predictions.^16)^ It offers high accuracy by effectively capturing complex relationships between variables and performing well with high-dimensional data.^16)^ By averaging the predictions of individual trees, it reduces overfitting and can handle missing values without significant performance loss. However, its main drawback is reduced interpretability compared to simpler models like decision tree analysis (DTA), making it difficult to understand the contribution of individual predictors. Careful hyperparameter tuning is essential to achieve optimal performance. This study conducted a random search over 100 trials to identify the parameters that yielded the highest AUC.

Our study underscores the importance of identifying high-risk patients who may not fully benefit from EVAR, even though it is less invasive than open repair. Despite its advantages, EVAR does not eliminate the risk of early postoperative mortality, particularly within the first 2 to 3 years. Previous studies have identified adverse predictors of mid-term mortality, such as advanced age, ischemic heart disease, and CKD, as well as favorable predictors, including small aneurysm size and statin use.^17,18)^ Our machine learning-based approach highlighted additional influential variables, particularly nutritional and immunological markers, which have been reported in more recent literature.^14,15,19–21)^ Specifically, preoperative malnutrition and sarcopenia, assessed by GNRI and controlling nutritional status score, are associated with increased mortality and major adverse cardiac and cerebrovascular events following EVAR. Therefore, these factors should be given more attention during preoperative evaluation to better assess patient risk and guide decision-making. Our findings suggest that tailored postoperative monitoring and management strategies are crucial for optimizing long-term survival after EVAR in patients with these risk factors.

Although many significant variables in the univariable analysis aligned with the top features identified by the random forest model, some discrepancies were noted. Univariable analysis assesses each factor independently, without considering inter-variable relationships or confounding,^6)^ whereas random forests evaluate all variables simultaneously, capturing nonlinear patterns and multicollinearity.^7)^ Thus, factors such as active cancer may appear less important if their effects overlap with others, while variables like statins may gain importance due to their unique predictive contribution.

Our random forest model demonstrated high predictive performance with an AUC of 0.91, an accuracy of 81.1%, a sensitivity of 81.6%, a specificity of 80.9%, and an F1 score of 0.66, supporting its clinical utility. These findings highlight the potential of the random forest model to assist in clinical decision-making and optimize patient management after EVAR. The results were validated using 5-fold cross-validation, a technique used to estimate the generalizability of the model, in which the dataset was divided into 5 subsets, with each serving as a test set while the model was trained on the remaining 4 subsets.^22)^ The average performance metrics across the folds were as follows: an AUC of 0.76 ± 0.10, an accuracy of 73.9 ± 5.8%, a sensitivity of 60.4 ± 1.9%, a specificity of 77.8 ± 0.7%, and an F1 score of 0.59 ± 0.17.

The findings of this study are almost consistent with the results of our previous DTA conducted on the same cohort.^11)^ Both the DTA and random forest models identified poor nutritional status, CKD, COPD, and octogenarian status as the primary risk factors. Although DTA provides an intuitive approach for stratifying patient risk groups, the random forest model demonstrated higher predictive accuracy. The random forest model achieved an accuracy of 81.1%, a sensitivity of 81.6%, and a specificity of 80.9%, whereas the DTA model had an accuracy of 68.7%, a sensitivity of 79.0%, and a specificity of 65.7%. The improved performance of the random forest model can be attributed to its ability to integrate multiple decision trees, reduce overfitting, and capture complex, nonlinear relationships within the dataset.

### Study limitations

There are several limitations to this study. First, the dataset used to train and validate the model is limited to a specific patient population, which may affect its generalizability to different cohorts. Second, while random forest provides high predictive accuracy, its interpretability remains a challenge, making it difficult to draw direct causal inferences from the model. Future research should focus on validating these findings in larger, multicenter cohorts and exploring ways to increase the interpretability of the model, such as using Shapley Additive Explanations analysis. Finally, further integration of clinical data and biomarkers could improve predictive accuracy and refine risk stratification models for patients undergoing EVAR.

## Conclusions

This study demonstrates that a machine learning-based random forest model effectively predicts 3-year survival after EVAR, achieving an AUC of 0.91 and an accuracy of 81.1%. Key factors identified include poor nutritional status, compromised immunity, CKD, octogenarian status, COPD, small aneurysm size, and statin use, which are crucial for preoperative risk assessment and personalized postoperative management. Random forest models offer superior predictive power compared to traditional approaches, while their interpretability remains a challenge. Future extensive research should validate these findings in larger cohorts and explore strategies to improve model transparency for clinical use.

## Declarations

### Funding

This research has not received any funding.

### Data availability

The data that support the findings of this study are not publicly available due to confidentiality restrictions but are available from the corresponding author upon reasonable request.

### Author contributions

All authors have read and approved the manuscript and are responsible for its content. Nishibe T: conception and design. Nishibe T, Iwasa T, Matsuda S: analysis and interpretation. Nishibe T, Kano M, Akiyama S, Fukuda S: data collection. Nishibe T, Nishibe M: writing the article. Nishibe T: critical revision of the article.

### Disclosure statement

The authors have no conflicts of interest to declare.
